# Six-month randomized, double-blind trial of transcranial direct current stimulation in mild Alzheimer's dementia: domain-specific cognitive and neuropsychiatric signals

**DOI:** 10.3389/fneur.2026.1749559

**Published:** 2026-02-23

**Authors:** YoungSoon Yang, Youngmin Huh, Kiwon Lee, Yong Tae Kwak

**Affiliations:** 1 Department of Neurology, Soonchunhyang University College of Medicine, Cheonan Hospital, Cheonan-si, Republic of Korea; 2 Ybrain Research Institute, Seongnam-si, Republic of Korea; 3 Department of Neurology, Hyoja Geriatric Hospital, Yongin-shi, Republic of Korea

**Keywords:** Alzheimer's disease, caregiver quality of life, cognition, home-based intervention, neuropsychiatric symptoms, transcranial direct current stimulation

## Abstract

**Background:**

Prefrontal transcranial direct current stimulation (tDCS) is a low-risk candidate intervention for cognitive enhancement in Alzheimer's disease (AD), but trial results are heterogeneous and often short-term. We evaluated the 26-week efficacy, safety, and family-level impact of home-based prefrontal tDCS in mild AD.

**Methods:**

In this randomized, double-blind, sham-controlled trial, 120 patients with mild AD were allocated to active (*n* = 59) or sham (*n* = 61) tDCS. The intention-to-treat (ITT) population included 106 participants (53 vs. 53) with post-baseline data; 66 (32 vs. 34) comprised the per-protocol (PP) set. The primary outcome was change in global cognition on the Korean Mini-Mental State Examination (K-MMSE). Secondary and exploratory outcomes included domain-specific cognition [e.g., Korean Boston Naming Test (K-BNT)], neuropsychiatric symptoms [Korean Neuropsychiatric Inventory (K-NPI)], patient quality of life (QoL-AD), and caregiver-reported family quality of life [Family Quality of Life–Dementia (FQoL-D)]. Adverse events (AEs) were systematically monitored.

**Results:**

K-MMSE declined slightly in both groups over 26 weeks (active Δ −0.53, sham Δ −0.15), with no significant between-group difference (*p* = 0.402). Most cognitive domains showed small, non-significant changes. In contrast, confrontation naming on the K-BNT favored active tDCS: in ITT analyses, naming performance was stable with active stimulation (Δ +0.51) but worsened with sham (Δ −2.32; *p* = 0.022), with a similar pattern in the PP set. K-NPI findings were inconsistent across analytic sets. Notably, FQoL-D declined in the active arm but improved in the sham arm in both ITT (Δ −2.19 vs. +1.94; *p* = 0.043) and PP analyses. Overall AE rates were similar; stimulation-site reactions were common but mild, and serious AEs were rare and deemed unrelated to tDCS.

**Conclusion:**

In mild AD, 26 weeks of home-based prefrontal tDCS did not improve global cognition vs. sham, although a modest benefit in confrontation naming was observed. The deterioration in caregiver-reported family quality of life highlights the need to weigh potential cognitive gains against family burden in long-term home-based neuromodulation.

**Clinical trial registration:**

Clinical Research Information Service (CRIS), KCT0005834.

## 1 Introduction

Alzheimer's disease (AD) is the most common cause of dementia worldwide and remains a major public health challenge in aging societies. Despite decades of research, currently available disease-modifying therapies provide only modest benefits and are not universally accessible, while symptomatic pharmacological options have limited effect sizes and may be constrained by tolerability issues (1–3). These limitations have driven growing interest in complementary and scalable non-pharmacological strategies that can be safely integrated with standard care, such as home-based neuromodulation.

Transcranial direct current stimulation (tDCS) is a non-invasive neuromodulation technique that delivers weak direct current through scalp electrodes to modulate cortical excitability and plasticity (4–6). When applied within guideline-recommended parameters, tDCS is generally well-tolerated and associated mainly with mild, transient adverse effects (7). Experimental and clinical studies suggest that anodal tDCS over the dorsolateral prefrontal cortex (DLPFC) can influence synaptic plasticity and large-scale networks implicated in cognition and emotion regulation (4, 8). In AD and mild cognitive impairment (MCI), small randomized and open-label trials have reported improvements in global cognition, memory, language, and executive function after repeated tDCS sessions, although effect sizes and durability have varied and most studies have involved relatively short treatment periods and modest sample sizes (9–12).

More recent work has begun to relate clinical outcomes of tDCS to disease-relevant biomarkers and network-level measures. In amyloid PET–positive mild AD, home-based bifrontal tDCS has been associated with improvements in language, verbal memory, attention, and frontal-executive functions, together with reduced plasma Aβ oligomerization tendency (13). In mild cognitive impairment (MCI), sequential anodal DLPFC tDCS has been associated with changes in resting-state functional segregation and integration even when group-level cognitive effects were subtle, suggesting that neuromodulation can induce measurable network plasticity in prodromal AD stages (14). In early AD, Im et al. conducted a 6-month randomized, double-blind, sham-controlled trial of home-based anodal tDCS targeting the prefrontal cortex (F3–F4 montage, 2 mA, 30 min per day). Active tDCS was associated with improvements in Mini-Mental State Examination and Boston Naming Test scores and attenuation of decline in left temporal FDG-PET metabolism compared with sham, providing proof-of-concept that long-term prefrontal tDCS may help stabilize cognitive trajectories and metabolic integrity in early disease (13). However, that study enrolled only 18 participants (11 active, seven sham), limiting precision and generalizability. While recent systematic reviews/meta-analyses suggest that tDCS may produce small, domain-specific cognitive effects in AD, they also point to heterogeneity and the need for well-controlled trials focused on clinically meaningful outcomes (15–17); similarly, network meta-analyses of non-invasive brain stimulation in AD and MCI call for larger, methodologically rigorous studies with longer follow-up and broader outcome assessment, including behavioral and caregiver-reported endpoints (11, 18). Converging evidence also indicates that the impact of tDCS in the AD spectrum is strongly modified by individual biological risk factors. Changes in white-matter microstructure and functional connectivity after tDCS have been shown to depend on amyloid status, APOE ε4 carriage, sex, and BDNF Val66Met polymorphism, with certain subgroups (e.g. APOE ε4 carriers, female Val/Val homozygotes) showing larger network-level responses (19, 20). These data support a precision-medicine framework in which stimulation effects are phenotype- and biomarker-dependent rather than uniform across patients.

Despite these developments, longer-term randomized data in dementia-stage AD remain sparse, and few trials have simultaneously evaluated global cognition, domain-specific cognitive outcomes, neuropsychiatric symptoms, and caregiver quality of life over extended periods. Detailed reporting of participant flow, adherence, and adverse events in home-based tDCS implementations has also been limited, making it challenging to assess real-world feasibility and safety (11, 21, 22). The present randomized, double-blind, sham-controlled trial was designed to address some of these gaps in a larger sample of patients with mild AD dementia. Building on the prior 6-month home-based tDCS trial in early AD (13), we implemented a 26-week stimulation protocol and a comprehensive battery spanning global and domain-specific cognition, daily functioning, neuropsychiatric symptoms, and both patient and caregiver quality of life. The primary outcome was 26-week change in the Korean Mini-Mental State Examination (K-MMSE), with secondary outcomes including other cognitive scales, functional measures, mood and behavioral indices, and caregiver-reported family quality of life.

We hypothesized that active prefrontal tDCS would attenuate global cognitive decline compared with sham over 26 weeks and might exert small, domain-specific effects in language and executive function, as well as measurable impact on caregiver-centered outcomes, while maintaining an acceptable long-term safety and tolerability profile.

## 2 Methods

### 2.1 Study design and participants

**Figure 1:**
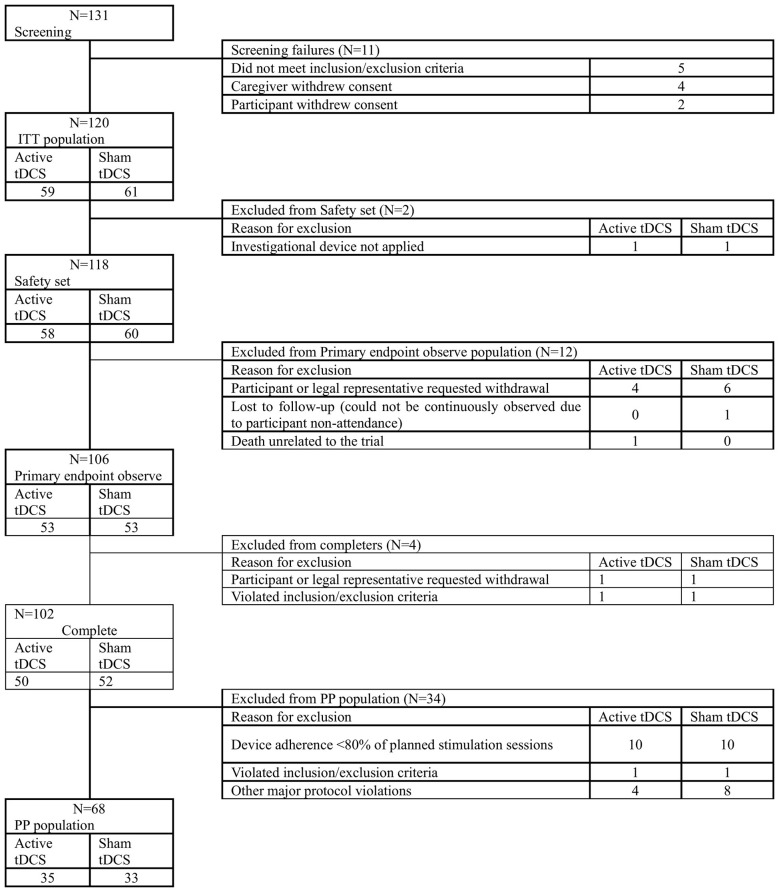
Participant disposition and analysis populations. Of 131 participants screened, 120 were randomized to active tDCS (*n* = 59) or sham tDCS (*n* = 61). The Safety set included all randomized participants who received at least one stimulation session. The ITT set included all randomized participants analyzed as randomized. The complete-case set for the primary endpoint comprised participants with observed baseline and week-26 K-MMSE data (*n* = 106). The PP set included participants who completed the 26-week double-blind phase without major protocol violations and with device adherence of at least 80% of planned sessions. Reasons for discontinuation and missing primary endpoint data are shown. tDCS, transcranial direct current stimulation; ITT, intention-to-treat; PP, per-protocol.

This was a parallel-group, multicenter, randomized, double-blind, sham-controlled clinical trial of prefrontal tDCS in patients with mild AD, conducted at seven university-affiliated hospitals in Korea. The protocol was approved by local institutional review boards and national regulatory authorities, and written informed consent was obtained from all participants or their legal representatives in accordance with the Declaration of Helsinki. Key inclusion criteria were aligned with contemporary diagnostic standards for AD dementia (1) and included age 55–90 years; a diagnosis of major neurocognitive disorder due to AD according to DSM-5/ICD-10 or equivalent clinical criteria; K-MMSE scores of 18–26; CDR global 0.5–1.0 and CDR-SB 0.5–4.0; stable treatment with acetylcholinesterase inhibitors and/or NMDA receptor antagonists for at least 3 months; and sufficient sensory abilities, with aids as needed, to complete neuropsychological testing. Major exclusion criteria comprised significant neurological or psychiatric comorbidities that could impair cognition, recent alcohol or substance misuse, uncontrolled systemic illness, contraindications to tDCS (e.g. cranial metal implants, skin disease at electrode sites), pregnancy, and participation in other interventional trials. A total of 120 participants were randomized (active tDCS, *n* = 59; sham, *n* = 61). The intention-to-treat (ITT) population comprised 106 participants (active *n* = 53, sham *n* = 53) with usable baseline and week-26 K-MMSE data for the primary outcome (Figure 1, Table 1).

**Table 1:** Baseline demographic and clinical characteristics of the ITT population.

**Characteristics**	**tDCS (*n* = 53)**	**Sham (*n* = 53)**	***p*-Value**
Age, year	73.64 ± 7.95	73.57 ± 6.31	0.957
Female gender (%)	37 (69.8%)	36 (67.9%)	1.000
Education year	9.7 ± 5.2	10.2 ± 5.7	0.641
K-MMSE	22.17 ± 2.40	22.21 ± 2.56	0.938
CDR	0.53 ± 0.12	0.55 ± 0.15	0.467
CDR-SB	2.66 ± 1.22	2.75 ± 0.97	0.662
SGDS	3.81 ± 3.49	3.68 ± 3.32	0.842
K-NPI	5.26 ± 7.96	6.09 ± 8.86	0.613

Data are presented as mean ± standard deviation unless otherwise indicated. Female gender is presented as percentage. K-MMSE, Korean version of the Mini-Mental State Examination; CDR, Clinical Dementia Rating; CDR-SB, CDR sum of boxes; SGDS, Short Geriatric Depression Scale; K-NPI, Korean version of the Neuropsychiatric Inventory.

p-Values were calculated using t-tests for continuous variables and χ^2^ or Fisher's exact tests for categorical variables, as appropriate.

### 2.2 Sample size and power

The target sample size was determined *a priori* on the basis of the expected between-group difference in change on the K-MMSE, the prespecified primary endpoint at 26 weeks. Previous tDCS trials in patients with mild Alzheimer's disease or related dementias have reported mean between-group differences in MMSE change of approximately 2.5–3.0 points over 6–12 months, with common standard deviations of around four points (9, 10, 15). Assuming a superiority margin of 0.21 points, a common standard deviation of 4.1, a one-sided α of 0.025 (equivalent to a two-sided α of 0.05), and 80% power to detect a between-group difference in 26-week K-MMSE change, we estimated that 88 evaluable patients (44 per group) would be required. Allowing for an anticipated dropout rate of 25%−30% over 6 months, we planned to randomize approximately 118–120 participants (about 59–60 per group). In the present trial, 120 participants were randomized and 106 (53 per group) were included in the ITT population.

### 2.3 Randomization and blinding

Participants were randomly assigned (1:1) to active or sham tDCS using a computer-generated schedule with concealed allocation. Randomization was implemented by an independent statistician or unblinded staff not involved in assessments. The tDCS device was pre-programmed so that the appearance and procedures were identical between active and sham modes, with sham stimulation mimicking the initial ramp-up sensation. Participants, caregivers, clinicians, raters, and primary data analysts remained blind to allocation throughout the trial. A formal assessment of blinding success (e.g., participant/caregiver/rater allocation guess) was not administered.

### 2.4 tDCS intervention

tDCS was administered with the YMS-201B device (Ybrain Inc., Seongnam, Korea). Following the international 10–20 EEG system, the anode was placed over the left dorsolateral prefrontal cortex (F3) and the cathode over the right dorsolateral prefrontal cortex (F4). Saline-soaked sponge electrodes were used to ensure stable conductivity and participant comfort. Stimulation intensity was set at 2 mA for 30 min per session. Sessions were delivered over the 26-week double-blind period according to a prespecified schedule of five sessions per week, self-administered at home at a convenient (non-fixed) time of day with encouragement to maintain a consistent routine. No standardized concurrent cognitive/language training task was prescribed during stimulation; in-session activities were not systematically recorded, and instructions were identical in the active and sham conditions. Participants were randomly assigned to Active-tDCS or Sham-tDCS. For sham, the device followed the same preparation and placement procedures, but current was discontinued after an initial ~30-s ramp, producing the brief tingling sensation used for blinding while avoiding sustained stimulation (23). To support feasibility and adherence, participants or caregivers received hands-on training in electrode positioning and device operation before treatment initiation. They were instructed to clean the skin and electrodes before/after sessions, record any discomfort or adverse events, and contact the study team with concerns. Regular follow-ups (on-site or remote) were conducted to monitor compliance and reinforce correct placement and procedures, in line with recommendations for remotely supervised/home-based tDCS implementations (21, 22). However, prespecified quantitative protocol-fidelity metrics (e.g., electrode-placement verification counts and remote-supervision frequency) were not systematically recorded for reporting. All device issues and adverse events were documented per protocol.

### 2.5 Outcomes and assessments

Neuropsychological and clinical assessments were conducted at baseline and week 26, with several scales additionally administered at week 13; the present report focuses on baseline and week-26 data. The primary outcome was the 26-week change in global cognition measured by K-MMSE. Pre-specified secondary outcomes included clinical severity and function [Clinical Dementia Rating (CDR) and Korean Instrumental Activities of Daily Living (K-IADL)]; memory and visuospatial function [Seoul Verbal Learning Test—Elderly (SVLT-E); Rey Complex Figure Test (RCFT)]; executive function and attention [Korean Stroop Word–Color Test (K-CWST); Digit Span Test (DST); Controlled Oral Word Association Test (COWAT)]; language [Korean Boston Naming Test (K-BNT)]; additional global cognition [Montreal Cognitive Assessment—Korean (MoCA-K)], with totals computed as the sum of item-level scores; and quality of life and behavioral symptoms [Quality of Life in Alzheimer's Disease (QoL-AD), using the dataset's designated total score field; Korean Neuropsychiatric Inventory (K-NPI), total computed as the sum of frequency × severity across domains; and Family Quality of Life–Dementia (FQoL-D), caregiver-reported total score]. For interpretation, lower scores indicated improvement for some measures (e.g., K-NPI, depression scales), whereas higher scores indicated improvement for others (e.g., K-MMSE, K-BNT, QoL-AD, FQoL-D). Safety outcomes included the incidence of any adverse event (AE), serious AEs, treatment-related AEs (defined as events with adverse event relationship (AEREL) codes indicating a definite, probable, or possible relationship to the investigational device), stimulation-site reactions (local symptoms such as pain, discomfort, burning sensation, itching, or skin discoloration at the forehead), and other non–stimulation-site AEs (e.g. headache, diarrhea, anorexia, COVID-19 infection, musculoskeletal pain). Safety events were coded and summarized for the Safety set (*n* = 118).

### 2.6 Analysis of populations and statistical methods

The primary estimand was the treatment-policy estimand at week 26, defined as the effect of assignment to active vs. sham tDCS on the primary outcome regardless of adherence, treatment discontinuation, or other intercurrent events. The per-protocol (PP) analysis was prespecified as supportive and targets a while-on-treatment/hypothetical estimand among participants meeting adherence and protocol-compliance criteria. Compliance (adherence) was calculated as the number of completed sessions divided by the prespecified planned sessions, based on device-recorded session completion; the ≥80% threshold was used only to define the PP population. Efficacy analyses were conducted in the ITT and PP populations as defined above; sample size varied slightly by endpoint. K-MMSE was the prespecified primary endpoint and the only outcome for which the trial was powered. All other cognitive domain measures, neuropsychiatric outcomes, and caregiver-reported quality-of-life measures were prespecified as secondary or exploratory outcomes and are interpreted as hypothesis-generating; therefore, *p*-values for these outcomes are reported nominally without formal multiplicity adjustment. Safety analyses were conducted in the ITT set. For each endpoint and analysis set (ITT, PP), we calculated baseline and week-26 means and standard deviations (SD) by group, the mean change (Δ = week 26—baseline), and SD of change. Between-group differences in change (active – sham) were tested using Welch two-sample *t*-tests. Two-sided *p*-values < 0.05 were considered statistically significant at the nominal level. Cohen's *d* effect sizes were computed as the difference in mean change divided by the pooled SD of change. To support an estimation-focused interpretation for key endpoints, we additionally report covariate-adjusted between-group differences (Active–Sham) with 95% confidence intervals and standardized effects (Hedges' *g*) for K-MMSE, K-BNT, and FQoL-D in Supplementary Table 4. The primary between-group comparisons at week 26 were based on observed outcomes (complete-case analysis).The primary efficacy analysis was conducted using a complete-case approach, restricted to participants with observed baseline and week-26 outcomes. As sensitivity analyses for missing data, we performed (i) a mixed-effects model for repeated measures (MMRM), which uses all available repeated measures under a missing-at-random assumption, and (ii) multiple imputation for missing week-26 outcomes. In the MMRM, treatment group, visit, and their interaction were included as fixed effects, with study site included as a fixed effect, and an unstructured within-participant covariance was assumed. For safety endpoints and categorical variables (e.g. AEs, sex), between-group comparisons used χ^2^ tests or Fisher's exact tests, as appropriate. In addition, for the primary endpoint we fitted an analysis of covariance (ANCOVA) model with 26-week change in K-MMSE as the dependent variable and treatment group (active vs. sham), baseline K-MMSE, and study site (seven-level factor) as independent variables, and we report the adjusted between-group mean difference with 95% confidence intervals from this model. These covariate-adjusted and longitudinal modeling strategies are widely used in large-sample database studies to improve precision and mitigate bias from baseline imbalance and incomplete follow-up (24, 25).

## 3 Results

### 3.1 Participants

A total of 120 participants were randomized to active tDCS (*n* = 59) or sham tDCS (*n* = 61). According to the disposition dataset, 102 participants (active *n* = 50, sham *n* = 52) completed the 26-week double-blind phase, whereas 18 discontinued early. Completion rates were similar between groups (84.7% in the active group and 85.2% in the sham group). The most common reason for dropout was withdrawal of consent by the participant or legal representative [active 7/59 (11.9%), sham 8/61 (13.1%)]; as shown in Figure 1, withdrawals were recorded categorically and more granular reasons for consent withdrawal were not systematically captured for reporting. Additional reasons included loss to follow-up in one sham participant and one death and one investigator-determined discontinuation in the active group. We report (a) trial completion of the double-blind phase (Figure 1) and (b) analysis sets defined by availability of outcome data. The complete-case analysis set was defined by observed baseline and week-26 K-MMSE data, which may include participants who discontinued treatment early but completed outcome assessments. For efficacy analyses, the ITT population included all randomized participants, analyzed according to randomized assignment. A complete-case set was defined as participants with observed baseline and week-26 K-MMSE data (*n* = 106; active *n* = 53, sham *n* = 53) and was used for sensitivity analyses. The per-protocol (PP) population comprised 66 participants (active *n* = 32, sham *n* = 34) who completed the 26-week double-blind phase without major protocol violations and with device adherence ≥80% of planned sessions (Figure 1, Tables 1 and 2).

**Table 2:** Outcomes at baseline (0 week) and 26 weeks by group.

**Endpoint**	**Active tDCS (*****n*** = **53)**			**Sham tDCS (*****n*** = **53)**			
	**0 week**	**26 week**	**thCS** Δ	**0 week**	**26 week**	**Sham** Δ	***p*** **(**Δ**)**
K-MMSE	22.17 ± 2.40	21.64 ± 3.71	−0.528	22.21 ± 2.56	22.06 ± 4.02	−0.151	0.402
CDR	0.53 ± 0.12	0.62 ± 0.22	0.094	0.55 ± 0.15	0.70 ± 0.54	0.151	0.481
K-IADL	5.23 ± 3.38	6.02 ± 4.32	0.792	5.13 ± 3.45	6.38 ± 4.40	1.245	0.338
SVLT-E	3.34 ± 1.43	3.26 ± 1.50	−0.075	3.72 ± 1.62	3.34 ± 1.54	−0.377	0.277
RCFT	3.37 ± 4.33	5.07 ± 6.89	1.698	3.21 ± 3.86	3.03 ± 3.98	−0.176	0.073
K-CWST	48.86 ± 28.25	50.59 ± 31.17	1.725	44.94 ± 27.48	42.58 ± 30.30	−2.360	0.253
DST	5.06 ± 1.23	5.19 ± 1.13	0.132	4.98 ± 1.22	5.00 ± 1.11	0.019	0.511
COWAT	9.77 ± 3.61	9.28 ± 3.59	−0.491	9.28 ± 4.61	8.74 ± 4.18	−0.547	0.923
K-BNT	37.45 ± 11.31	37.96 ± 10.78	+0.51	35.23 ± 13.15	32.91 ± 14.00	−2.32	0.022
SGDS	3.81 ± 3.49	3.89 ± 3.71	0.075	3.68 ± 3.32	4.09 ± 3.72	0.415	0.548
GDS	3.45 ± 0.50	3.49 ± 0.54	0.038	3.47 ± 0.58	3.58 ± 0.66	0.113	0.310
MoCA-K	16.96 ± 4.04	16.28 ± 5.41	−0.679	16.06 ± 4.87	15.55 ± 5.55	−0.509	0.752
QoL-AD	32.23 ± 5.91	32.04 ± 6.72	−0.189	32.17 ± 5.99	32.40 ± 5.92	0.226	0.670
NPI Total	5.26 ± 7.96	5.62 ± 7.25	0.358	6.09 ± 8.86	9.81 ± 14.58	3.717	0.065
FQoL-D	91.83 ± 7.93	89.64 ± 13.74	−2.189	94.94 ± 9.54	96.89 ± 9.82	1.943	0.043

Values are mean ± SD. Δ is computed as group mean at 26 weeks minus group mean at baseline. p is Welch t-test for Δ difference between groups. p-Values for secondary/exploratory outcomes are nominal and are not adjusted for multiplicity; corresponding 95% confidence intervals and standardized effect sizes are provided to support estimation-focused interpretation.

K-MMSE, Korean Mini-Mental State Examination; CDR, Clinical Dementia Rating; K-IADL, Korean Instrumental Activities of Daily Living; SVLT-E, Seoul Verbal Learning Test–Elderly; RCFT, Rey Complex Figure Test; K-CWST, Korean Color-Word Stroop Test; DST, Digit Span Test; COWAT, Controlled Oral Word Association Test; K-BNT, Korean Boston Naming Test; SGDS, Short Geriatric Depression Scale; GDS, Global Deterioration Scale; MoCA-K, Montreal Cognitive Assessment–Korean; QoL-AD, Quality of Life in Alzheimer's Disease; K-NPI, Korean Neuropsychiatric Inventory; FQoL-D, Family Quality of Life–Dementia; tDCS, transcranial direct current stimulation.

### 3.2 Baseline demographics characteristics

Baseline demographic and clinical characteristics of the ITT population are summarized in Table 1. Mean age was 73.6 ± 7.9 years in the active tDCS group and 73.6 ± 6.3 years in the sham group, and approximately two-thirds of participants were female (69.8% active vs. 67.9% sham). Years of education were comparable between groups (9.7 ± 5.2 vs. 10.2 ± 5.7). Baseline cognitive and clinical severity were typical for mild AD dementia and well-balanced across arms: mean K-MMSE scores were 22.17 ± 2.40 (active) and 22.21 ± 2.56 (sham); mean CDR global scores were 0.53 ± 0.12 and 0.55 ± 0.15, respectively; and mean CDR-SB scores were 2.66 ± 1.22 and 2.75 ± 0.97. Depressive symptoms (SGDS) and neuropsychiatric burden (K-NPI) were modest overall (SGDS 3.81 ± 3.49 vs. 3.68 ± 3.32; K-NPI 5.26 ± 7.96 vs. 6.09 ± 8.86) with no statistically significant between-group differences (all *p* ≥ 0.47; Table 1). Baseline neuropsychological performance (Table 2) indicated broadly mild impairment across multiple domains (memory, executive/attention, and language), with overall modest neuropsychiatric burden at study entry (K-NPI totals).

### 3.3 Global cognition

Global cognition, as measured by the K-MMSE, declined modestly in both groups over 26 weeks (Table 2). In the ITT population, mean K-MMSE change was −0.53 points in the active tDCS group (22.17 ± 2.40 to 21.64 ± 3.71) and −0.15 points in the sham group (22.21 ± 2.56 to 22.06 ± 4.02); the between-group difference in change was not statistically significant (*p* = 0.402). MoCA-K scores showed a similar pattern, with small declines in both groups (active Δ −0.68, sham Δ −0.51) and no evidence of a differential treatment effect (*p* = 0.752). Secondary and exploratory outcomes are presented to characterize domain-specific signals; results are interpreted cautiously in light of multiple comparisons, with emphasis on effect sizes and 95% confidence intervals rather than statistical significance alone.

### 3.4 Domain-specific cognitive outcomes

Domain-specific cognitive outcomes are summarized in Table 2 and Supplementary Table 1. Episodic verbal memory (SVLT-E total recall) declined slightly in both groups (active Δ −0.08, sham Δ −0.38; *p* = 0.277), and measures of executive function and attention (K-CWST, DST, COWAT) showed only small, non-significant changes with substantial overlap between groups (all *p* > 0.17). Visuospatial performance on the RCFT showed a numerical divergence between groups. In the ITT set, RCFT scores increased in the active group (mean Δ +1.70) but were essentially stable or slightly lower in the sham group (mean Δ −0.18; *p* = 0.073). A similar, non-significant trend was observed in the PP population (active Δ +1.77 vs. sham Δ −0.24; *p* = 0.132; Supplementary Table 2). In contrast, confrontation naming on the K-BNT showed a small but statistically significant between-group difference favoring active tDCS. In the ITT population, the active group was relatively stable or showed a slight improvement (37.45 ± 11.31 to 37.96 ± 10.78; mean Δ +0.51), whereas the sham group exhibited a decline (35.23 ± 13.15 to 32.91 ± 14.00; mean Δ −2.32), yielding a nominally significant between-group difference in change (*p* = 0.022; Table 2). In the PP set, this pattern was maintained, with active participants again remaining stable or slightly improved (Δ +0.65) and sham participants showing decline (Δ −2.03; *p* = 0.043; Supplementary Table 2). SGDS and patient-reported quality of life (QoL-AD) demonstrated minimal mean changes in both groups and no meaningful between-group differences (all *p* ≥ 0.31; Table 2, Supplementary Table 2).

### 3.5 Neuropsychiatric symptoms

Neuropsychiatric symptoms, indexed by the K-NPI total score, showed numerically smaller worsening in the active tDCS group than in the sham group in the ITT analysis (Table 2). In the ITT population, mean K-NPI change was +0.36 points in the active group and +3.72 points in the sham group (*p* = 0.065), suggesting a trend toward relatively more stable behavioral symptoms under active tDCS, although the result did not reach conventional significance. In the PP population, however, the direction of effect was reversed: active participants showed a mean increase of +5.03 points, whereas sham participants showed a slight decrease (Δ −0.47; *p* = 0.028; Supplementary Table 2). Given these discrepant patterns between ITT and PP analyses, the modest sample sizes, and the lack of multiplicity correction, K-NPI findings should be interpreted cautiously as exploratory and analytically unstable rather than as definitive evidence of benefit or harm.

### 3.6 Caregiver quality of life

Caregiver-reported family quality of life, assessed by the FQoL-D, showed a consistent pattern favoring the sham group rather than active tDCS (Table 2, Supplementary Tables 1, 2). In the ITT population, mean FQoL-D scores declined in the active group (91.83 ± 7.93 to 89.64 ± 13.74; mean Δ −2.19) but improved in the sham group (94.94 ± 9.54 to 96.89 ± 9.82; mean Δ +1.94), resulting in a nominally significant between-group difference in change (*p* = 0.043; Table 2). In the PP set, this pattern was even more pronounced: active participants showed a mean decline of −2.97 points, whereas sham participants improved by +3.18 points (*p* = 0.042; Supplementary Table 2). Item-level analyses (Supplementary Table 3) indicated that this divergence was not driven by a single question but by small, consistent differences across multiple items related to emotional support, access to medical care, time for caregivers' own activities, and availability of help in special situations. For example, sham caregivers reported improvements in “having time for their own hobbies” (item 8; Δ +0.09 vs. −0.28, *p* = 0.016) and “receiving satisfactory emotional support” (item 10; Δ +0.15 vs. −0.23, *p* = 0.013), whereas active caregivers tended to report stable or slightly worse scores on these items.

### 3.7 Sensitivity analysis

Sensitivity analyses using an MMRM and multiple imputation for missing week-26 K-MMSE yielded results consistent with the primary complete-case analysis; no conclusions regarding statistical significance were changed (Supplementary Table 1).

### 3.8 Safety

Adverse events during the 26-week double-blind period are summarized in Table 3. Any AE occurred in 33/53 participants (62.3%) in both sham and active groups. Treatment-related AEs, all of which were stimulation-site reactions at the forehead, were reported in 24/53 (45.3%) sham participants and 31/53 (58.5%) active participants (*p* = 0.243). Stimulation-site reactions included pain, discomfort, burning sensation, itching, and skin discoloration; these events were generally mild and transient. Other (non–stimulation-site) AEs such as headache, diarrhea, anorexia, COVID-19 infection, and musculoskeletal pain—occurred in ≤ 2 participants ( ≤ 3.8%) per event type and were numerically more frequent in the sham group [13/53 (24.5%)] than in the active group [6/53 (11.3%), *p* = 0.127]. Serious AEs were infrequent: they occurred in 3/53 (5.7%) sham participants and in none of the active participants (*p* = 0.243). No serious AE was judged to be related to the investigational device. Detailed reasons for dropout are summarized in Table 4.

**Table 3:** Adverse events during the 26-week double-blind period (ITT population, *n* = 106).

**Adverse event**	**tDCS *n*/*N* (%)**	**Sham *n*/*N* (%)**	***p*-Value**
Any AE	33/53 (62.3%)	33/53 (62.3%)	1.000
Any serious AE^a^	3/53 (5.7%)	0/53 (0.0%)	0.243
Treatment-related AE^b^	24/53 (45.3%)	31/53 (58.5%)	0.243
Stimulation-site reactions^c^	24/53 (45.3%)	31/53 (58.5%)	0.243
Other AEs (non–stimulation-site)^d^	13/53 (24.5%)	6/53 (11.3%)	0.127

^a^Serious adverse events included ulcerative keratosis, severe diarrhea, and COVID-19 infection and all judged unrelated to the investigational device.

^b^Treatment-related AEs were defined as events with AEREL codes indicating a definite, probable, or possible relationship to the investigational device (AEREL = 1–3); in this dataset, all such events were stimulation-site reactions at the forehead.

^c^Stimulation-site reactions included local symptoms at the stimulation site such as pain, discomfort, burning sensation, itching, and skin discoloration of the forehead.

^d^Other AEs (non–stimulation-site) included systemic or non-local events such as headache, diarrhea, anorexia, COVID-19 infection, and musculoskeletal pain, each occurring in ≤ 2 participants ( ≤ 3.8%) in either group.

**Table 4:** Reasons for dropout during the 26-week double-blind phase.

**Adverse event**	**tDCS *N* (%)**	**Sham *N* (%)**	***p*-Value**
Participant/legal representative requested withdrawal	7 (11.9%)	8 (13.1%)	1.000
Lost to follow-up/cannot be observed	0 (0.0%)	1 (1.6%)	1.000
Death unrelated to trial	1 (1.7%)	0 (0.0%)	0.492
Investigator decision (continuation inappropriate)	1 (1.7%)	0 (0.0%)	0.492
Total dropout, *n* (%)	9 (15.3%)	9 (14.8%)	1.000

Values are number of participants (%). Two-sided p-values are from Fisher's exact test comparing proportions between groups.

## 4 Discussion

This 26-week randomized, double-blind, sham-controlled trial in mild Alzheimer's dementia did not demonstrate a clear advantage of prefrontal tDCS over sham on the primary outcome, the K-MMSE, with modest and comparable declines in both groups. Across secondary and exploratory outcomes, we observed a heterogeneous pattern of small, domain-specific effects rather than a uniform cognitive or behavioral benefit. This emphasis on heterogeneity is consistent with large-cohort, reproducible studies that model multi-domain neural/cognitive/psychopathological dimensions and identify subtype structure. Such work highlights why interventions may yield small, domain-specific signals in unselected samples rather than uniform benefits across outcomes (26–28). In particular, confrontation naming on the K-BNT improved slightly in the active tDCS group but declined in the sham group, yielding a statistically significant between-group difference, whereas most other cognitive and functional measures showed minimal, non-significant changes. We therefore interpret these domain-specific findings primarily based on effect sizes and 95% confidence intervals (Supplementary Table 4), rather than nominal *p*-values alone. Neuropsychiatric symptom trajectories differed between ITT and PP analyses, and caregiver-reported family quality of life (FQoL-D) consistently worsened in the active arm relative to sham, despite the identical nominal treatment schedule. Together, these results indicate that long-term, home-based prefrontal tDCS is feasible and well-tolerated in mild AD, but its clinical impact appears modest, domain-dependent, and potentially sensitive to adherence and analytic choices.

Our findings partly converge with and partly diverge from prior tDCS studies in the AD spectrum. The 6-month home-based trial by Im et al. reported improvements in global cognition and naming, as well as attenuation of temporal hypometabolism, in a small sample of early AD patients receiving prefrontal tDCS compared with sham (15). In our larger mild dementia cohort, we did not detect a benefit of tDCS on K-MMSE–defined global cognition, but we did observe a small, statistically significant advantage in confrontation naming (K-BNT) favoring active tDCS and a numerical trend toward greater change on the RCFT in the active arm. These results are compatible with the notion that prefrontal stimulation may preferentially modulate language and visuoconstructive networks in some patients, even when global screening measures remain largely unchanged. Mechanistically, the DLPFC is embedded in fronto-temporal control networks relevant to lexical retrieval and selection, which may help explain why a small signal emerged in confrontation naming despite minimal effects on global cognition (29). However, the effect sizes were small, confidence intervals overlapped, and no other cognitive domains—including episodic memory, executive function, attention, and MoCA-K—showed robust between-group differences, underscoring that any tDCS-related cognitive gains in dementia-stage AD are likely to be subtle.

The neuropsychiatric and caregiver-focused outcomes provide additional, and somewhat sobering, context. In the ITT set, K-NPI total scores tended to worsen less in the active tDCS group than in the sham group, with a trend-level *p*-value, whereas in the PP set K-NPI increased more in the active arm and remained stable or slightly improved in the sham arm. This analytic instability suggests that neuropsychiatric effects were modest and highly sensitive to missing data, adherence, and population definition. By contrast, FQoL-D showed a more consistent pattern: caregiver-reported family quality of life declined in the active tDCS arm and improved in the sham arm in both ITT and PP analyses, with nominally significant between-group differences. Because higher FQoL-D scores reflect better family and caregiver quality of life, these findings indicate that, under the conditions of this trial, home-based prefrontal tDCS was associated with a small but unfavorable impact on caregivers' perceived family wellbeing relative to sham. This may reflect caregiver burden related to the home-based stimulation routine (e.g., time burden, perceived responsibility, increased monitoring demands, or caregiver stress). Because caregiver contact intensity and protocol-related time burden were not formally quantified, this interpretation remains exploratory. Item-level analyses of FQoL-D (Supplementary Table 3) suggest that this divergence was not confined to a single domain but driven by multiple facets of family functioning and support. Sham caregivers tended to report relative stability or improvement in items related to accepting differences of opinion, time for personal hobbies, having someone to turn to in special situations, receiving emotional support, and accessing medical care, whereas active-arm caregivers more often reported declines in these areas. Importantly, sham participants and caregivers followed the same nominal 26-week home-based schedule and completed the same number of 30-min sessions, so simple time burden or organizational demands are unlikely to fully explain the active–sham discrepancy in FQoL-D. Instead, the higher frequency of stimulation-site reactions in the active arm, subtle “micro-unblinding” due to stronger sensations during active sessions, or more frequent contacts with healthcare services may have altered family dynamics and perceived burden (30), even in the absence of large between-group differences in global cognition. These hypotheses remain speculative, but they underscore that neuromodulation protocols should be evaluated not only for patient-level efficacy but also for their net impact on caregivers.

The broader neuromodulation literature supports a view of tDCS effects in AD as small, task-specific, and strongly modified by individual disease biology rather than uniformly beneficial. Network-level and biomarker studies have shown that changes in functional connectivity, white-matter microstructure, and even amyloid-related measures after tDCS depend on factors such as amyloid status, APOE ε4 carriage, BDNF polymorphism, and sex (13, 14, 19, 20, 31). Within this precision-medicine framework, our unselected mild AD sample likely included patients with heterogeneous pathology and residual plasticity, diluting any subgroup-specific benefits that might have been detectable in a more targeted population. The small but significant K-BNT effect and the trend in RCFT may reflect such phenotype-dependent responsiveness of language and fronto-parietal networks, while the absence of clear advantages on global measures suggests that prefrontal tDCS alone is insufficient to counteract medial temporal and parietal degeneration that drives overall cognitive decline (1, 32).

From a clinical perspective, the discrepancy between modest or inconsistent patient-level benefits and the unfavorable caregiver quality-of-life signal is particularly important. Behavioral and psychological symptoms of dementia are major determinants of caregiver burden, institutionalization, and healthcare costs (33), and family-centered outcomes are increasingly recognized as critical endpoints in dementia trials. In our study, the combination of small, unstable effects on K-NPI and consistent worsening of FQoL-D in the active arm suggests that, at least in this implementation, long-term home-based tDCS may not reliably translate into net gains for families. This does not preclude the possibility that optimized stimulation parameters, better targeting, or biomarker-guided patient selection could yield more favorable benefit–burden profiles, but it does argue against assuming caregiver advantages in the absence of direct evidence.

This trial has several strengths. It extends prior tDCS work by using a 26-week double-blind, sham-controlled design in a comparatively large mild AD sample and by implementing a monitored home-based protocol that approximates real-world practice. The comprehensive outcome battery—including global and domain-specific cognition, functional measures, neuropsychiatric symptoms, and both patient- and caregiver-reported quality of life—allows a more nuanced evaluation of tDCS effects than reliance on global scales alone. Detailed reporting of participant disposition, adherence, and AEs supports a transparent assessment of feasibility and safety, and similar overall AE rates between active and sham arms reinforce the tolerability of long-term prefrontal tDCS within recommended parameters (4–7).

At the same time, several limitations must be acknowledged. Multiple secondary and exploratory endpoints were assessed without formal multiplicity correction; therefore, nominal *p*-values may overstate evidence for domain-specific effects, and these findings should be interpreted cautiously as hypothesis-generating. Because multiple secondary outcomes were examined, domain-specific findings should be considered exploratory and hypothesis-generating; the observed signals warrant replication in adequately powered trials with prespecified multiplicity strategies. Sample sizes for some scales, particularly K-NPI and FQoL-D, were modest, increasing uncertainty around effect estimates and contributing to discrepancies between ITT and PP analyses. We did not obtain detailed biomarker data (e.g. amyloid PET, APOE genotype, BDNF polymorphism), precluding moderator analyses that might identify subgroups with clearer benefit or harm. Completion and overall adverse-event rates were comparable between groups, which does not suggest marked differential tolerability. However, formal assessment of blinding success (allocation-guess questionnaires) and prespecified quantitative protocol-fidelity metrics (e.g., electrode-placement verification frequency and remote-supervision intensity) were not collected; therefore, subtle unblinding and implementation variability cannot be fully excluded. Finally, we did not include quantitative neuroimaging or other mechanistic measures (e.g., MRI-based atrophy metrics, network imaging, or electrophysiology), so inferences about circuit-level effects of tDCS in this cohort remain speculative.

Overall, home-based prefrontal tDCS without a standardized concurrent behavioral intervention showed limited effects on global cognition, with exploratory domain-specific signals (notably naming); larger, well-controlled trials—particularly those pairing stimulation with prespecified cognitive/language training—are needed to clarify clinical benefit and potential augmentation of behavioral treatment effects, including clinically meaningful slowing of decline.

## 5 Conclusion

In this 26-week randomized, double-blind, sham-controlled trial in mild Alzheimer's dementia, prefrontal tDCS did not improve global cognition compared with sham. Domain-level effects were small and inconsistent, and caregiver-reported family quality of life worsened in the active arm. tDCS in dementia-stage AD should therefore be pursued, if at all, within biomarker-informed, carefully monitored trials that include caregiver outcomes as core endpoints.

## Data Availability

The raw data supporting the conclusions of this article will be made available by the authors, without undue reservation.

## References

[1] Scheltens P Blennow K Breteler MMB de Strooper B Frisoni GB Salloway S . Alzheimer's disease. Lancet. (2016) 388:505–17. doi: 10.1016/S0140-6736(15)01124-1 26921134

[2] Bikson M Grossman P Thomas C Zannou AL Jiang J Adnan T . Safety of transcranial direct current stimulation: evidence based update 2016. Brain Stimul. (2016) 9:641–61. doi: 10.1016/j.brs.2016.06.004 27372845 PMC5007190

[3] Antal A Alekseichuk I Bikson M Brockmöller J Brunoni AR Chen R . Low intensity transcranial electric stimulation: safety, ethical, legal, regulatory and application guidelines. Clin Neurophysiol. (2017) 128:1774–809. doi: 10.1016/j.clinph.2017.06.001 28709880 PMC5985830

[4] Lefaucheur JP Antal A Ayache SS Benninger DH Brunelin J Cogiamanian F . Evidence-based guidelines on the therapeutic use of transcranial direct current stimulation (tDCS). Clin Neurophysiol. (2017) 128:56–92. doi: 10.1016/j.clinph.2016.10.087 27866120

[5] Brunoni AR Amadera J Berbel B Volz MS Rizzerio BG Fregni F . A systematic review on reporting and assessment of adverse effects associated with transcranial direct current stimulation. Int J Neuropsychopharmacol. (2011) 14:1133–45. doi: 10.1017/S1461145710001690 21320389

[6] Boggio PS Ferrucci R Mameli F Martins D Martins O Vergari M . Prolonged visual memory enhancement after direct current stimulation in Alzheimer's disease. Brain Stimul. (2012) 5:223–30. doi: 10.1016/j.brs.2011.06.006 21840288

[7] Khedr EM Gamal NF Abo El-Fetoh N Khalifa H Ahmed EM Ali AM . A double-blind randomized clinical trial on the efficacy of cortical direct current stimulation for the treatment of Alzheimer's disease. Front Aging Neurosci. (2014) 6:275. doi: 10.3389/fnagi.2014.00275 25346688 PMC4191219

[8] Khedr EM Salama RH Abdel Hameed M Abo Elfetoh N Seif P. Therapeutic role of transcranial direct current stimulation in Alzheimer disease patients: double-blind, placebo-controlled clinical trial. Neurorehabil Neural Repair. (2019) 33:384–94. doi: 10.1177/1545968319840285 30940012

[9] Xu Y Qiu Z Zhu J Liu J Wu J Tao J. The modulation effect of non-invasive brain stimulation on cognitive function in patients with mild cognitive impairment: a systematic review and meta-analysis of randomized controlled trials. BMC Neurosci. (2019) 20:2. doi: 10.1186/s12868-018-0484-2 30602377 PMC6317253

[10] Park J Chung K Oh Y Kim KJ Kim CO Park JY. Effect of home-based transcranial direct current stimulation on cognitive function in patients with mild cognitive impairment: a two-week intervention. Yonsei Med J. (2024) 65:341–7. doi: 10.3349/ymj.2023.0430 38804028 PMC11130587

[11] Kim J Yang Y. Alterations in cognitive function and blood biomarkers following transcranial direct current stimulation in patients with amyloid positron emission tomography-positive Alzheimer's disease: a preliminary study. Front Neurosci. (2023) 17:1327886. doi: 10.3389/fnins.2023.1327886 38178837 PMC10765986

[12] Kang DW Wang SM Kim TY Kim D Na HR Kim NY . Impact of transcranial direct current stimulation on cognitive function, brain functional segregation, and integration in patients with mild cognitive impairment according to amyloid-beta deposition and APOE ε4-allele: a pilot study. Brain Sci. (2021) 11:772. doi: 10.3390/brainsci11060772 34200847 PMC8230518

[13] Im JJ Jeong H Bikson M Woods AJ Unal G Oh JK . Effects of 6-month at-home transcranial direct current stimulation on cognition and cerebral glucose metabolism in Alzheimer's disease. Brain Stimul. (2019) 12:1222–8. doi: 10.1016/j.brs.2019.06.003 31196835 PMC6703942

[14] Chu CS Li CT Brunoni AR Yang FC Tseng PT Tu YK . Cognitive effects and acceptability of non-invasive brain stimulation on Alzheimer's disease and mild cognitive impairment: a component network meta-analysis. J Neurol Neurosurg Psychiatry. (2021) 92:195–203. doi: 10.1136/jnnp-2020-323870 33115936 PMC7841477

[15] Kang DW Wang SM Um YH Kim S Kim T Kim D . Impact of transcranial direct current stimulation on white matter microstructure integrity in mild cognitive impairment patients according to effect modifiers as risk factors for Alzheimer's disease. Front Aging Neurosci. (2023) 15:1234086. doi: 10.3389/fnagi.2023.1234086 37744398 PMC10517264

[16] Kang DW Wang SM Um YH Kim S Kim T Kim D . Transcranial direct current stimulation and neuronal functional connectivity in MCI: role of individual factors associated to Alzheimer's disease. Front Psychiatry. (2024) 15:1428535. doi: 10.3389/fpsyt.2024.1428535 39224475 PMC11366601

[17] Gandiga PC Hummel FC Cohen LG. Transcranial DC stimulation (tDCS): a tool for double-blind sham-controlled clinical studies in brain stimulation. Clin Neurophysiol. (2006) 117:845–50. doi: 10.1016/j.clinph.2005.12.003 16427357

[18] Charvet LE Kasschau M Datta A Knotkova H Stevens MC Alonzo A . Remotely-supervised transcranial direct current stimulation (tDCS) for clinical trials: guidelines for technology and protocols. Front Syst Neurosci. (2015) 9:26. doi: 10.3389/fnsys.2015.00026 25852494 PMC4362220

[19] Charvet LE Shaw MT Bikson M Woods AJ Knotkova H. Supervised transcranial direct current stimulation (tDCS) at home: a guide for clinical research and practice. Brain Stimul. (2020) 13:686–93. doi: 10.1016/j.brs.2020.02.011 32289698

[20] Kang DW Wang SM Um YH Kim S Kim T Kim D . Effects of transcranial direct current stimulation on cognition in MCI with Alzheimer's disease risk factors using Bayesian analysis. Sci Rep. (2024) 14:18818. doi: 10.1038/s41598-024-67664-9 39138281 PMC11322558

[21] Freitas C Mondragón-Llorca H Pascual-Leone A. Noninvasive brain stimulation in Alzheimer's disease: systematic review and perspectives for the future. Exp Gerontol. (2011) 46:611–27. doi: 10.1016/j.exger.2011.04.001 21511025 PMC3589803

[22] Cummings JL Mega M Gray K Rosenberg-Thompson S Carusi DA Gornbein J. The neuropsychiatric inventory: comprehensive assessment of psychopathology in dementia. Neurology. (1994) 44:2308–14. doi: 10.1212/WNL.44.12.2308 7991117

[23] Kessler SK Turkeltaub PE Benson JG Hamilton RH. Differences in the experience of active and sham transcranial direct current stimulation. Brain Stimul. (2012) 5:155–62. doi: 10.1016/j.brs.2011.02.007 22037128 PMC3270148

[24] Wu X Zhang K Kuang N Kong X Cao M Lian Z . Developing brain asymmetry shapes cognitive and psychiatric outcomes in adolescence. Nat Commun. (2025) 16:6325. doi: 10.1038/s41467-025-61785-z 40634360 PMC12241635

[25] Liu Y Peng S Wu X Liu Z Lian Z Fan H . Neural, cognitive and psychopathological signatures of a prosocial or delinquent peer environment during early adolescence. Dev Cogn Neurosci. (2025) 73:101566. doi: 10.1016/j.dcn.2025.101566 40359598 PMC12140950

[26] Yu G Wu X Liu Z Shi M Fan H Liu Y . Genetic influence and neural pathways underlying the dose-response relationships between wearable-measured physical activity and mental health in adolescence. Psychiatry Res. (2025) 349:116503. doi: 10.1016/j.psychres.2025.116503 40347767

[27] Hertrich I Dietrich S Blum C Ackermann H. The role of the dorsolateral prefrontal cortex for speech and language processing. Front Hum Neurosci. (2021) 15:645209. doi: 10.3389/fnhum.2021.645209 34079444 PMC8165195

[28] Yu G Liu Z Wu X Becker B Zhang K Fan H . Common and disorder-specific cortical thickness alterations in internalizing, externalizing and thought disorders during early adolescence: an adolescent brain and cognitive development study. J Psychiatry Neurosci. (2023) 48:E345–56. doi: 10.1503/jpn.220202 37673436 PMC10495167

[29] Kuang N Liu Z Yu G Wu X Becker B Fan H . Neurodevelopmental risk and adaptation as a model for comorbidity among internalizing and externalizing disorders: genomics and cell-specific expression enriched morphometric study. BMC Med. (2023) 21:291. doi: 10.1186/s12916-023-02920-9 37542243 PMC10403847

[30] Majdi A van Boekholdt L Sadigh-Eteghad S Mc Laughlin M. A systematic review and meta-analysis of transcranial direct-current stimulation effects on cognitive function in patients with Alzheimer's disease. Mol Psychiatry. (2022) 27:2000–9. doi: 10.1038/s41380-022-01444-7 35115703

[31] Hou Y Liu F Su G Tu S Lyu Z. Systematic review and meta-analysis of transcranial direct current stimulation (tDCS) for global cognition in mild cognitive impairment and Alzheimer's disease. Geriatr Nurs. (2024) 59:261–70. doi: 10.1016/j.gerinurse.2024.07.013 39089145

[32] Frisoni GB Aho E Brayne C Ciccarelli O Dubois B Fox NC . Alzheimer's disease outlook: controversies and future directions. Lancet. (2025) 406:1424–42. doi: 10.1016/S0140-6736(25)01389-3 40997840

[33] Wu CK Fuh JL. A 2025 update on treatment strategies for the Alzheimer's disease spectrum. J Chin Med Assoc. (2025) 88:495–502. doi: 10.1097/JCMA.0000000000001252 40442885 PMC12637128

