# Supplementary material for: Six-month randomized, double-blind trial of transcranial direct current stimulation in mild Alzheimer's dementia: domain-specific cognitive and neuropsychiatric signals
Source: Front Neurol. 2026 Feb 23;17:1749559. doi: 10.3389/fneur.2026.1749559 (PMC12967943; doi:10.3389/fneur.2026.1749559)
Supplement: Supplementary file 1 [file Table_1.docx]

**Supplementary Table 1. MMRM-Based Least-Squares Mean Estimates of Clinical Outcomes at Baseline and Week 26 and Treatment-by-Visit Interaction Effects (All Randomized Participants, N = 120)**

| Endpoint | Active tDCS (N=59) | | | Sham tDCS(n=61) | | |  |
| --- | --- | --- | --- | --- | --- | --- | --- |
|  | 0 week (LSM±SE) | 26 week (LSM±SE) | tDCS Δ | 0 week (LSM±SE) | 26 week (LSM±SE) | Sham Δ | p (Δ) |
| K-MMSE | 22.25 ± 0.38 | 21.63 ± 0.39 | -0.622 | 22.03 ± 0.38 | 21.88 ± 0.39 | -0.151 | 0.284 |
| CDR | 0.53 ± 0.04 | 0.62 ± 0.04 | 0.095 | 0.54 ± 0.04 | 0.71 ± 0.04 | 0.166 | 0.341 |
| K-IADL | 5.32 ± 0.43 | 6.18 ± 0.44 | 0.860 | 5.38 ± 0.42 | 6.59 ± 0.44 | 1.217 | 0.437 |
| SVLT-E | 3.41 ± 0.18 | 3.30 ± 0.19 | -0.107 | 3.64 ± 0.18 | 3.30 ± 0.19 | -0.336 | 0.390 |
| RCFT | 3.24 ± 0.59 | 4.90 ± 0.60 | 1.661 | 3.18 ± 0.57 | 3.05 ± 0.61 | -0.132 | 0.072 |
| K-CWST | 49.68 ± 3.61 | 50.64 ± 3.65 | 0.955 | 44.09 ± 3.51 | 41.90 ± 3.63 | -2.190 | 0.365 |
| DST | 5.08 ± 0.14 | 5.20 ± 0.15 | 0.115 | 5.00 ± 0.14 | 5.02 ± 0.15 | 0.020 | 0.566 |
| COWAT | 9.88 ± 0.51 | 9.36 ± 0.52 | -0.523 | 9.16 ± 0.50 | 8.67 ± 0.52 | -0.493 | 0.957 |
| K-BNT | 37.31 ± 1.53 | 37.76 ± 1.54 | 0.446 | 33.74 ± 1.50 | 31.61 ± 1.53 | -2.127 | 0.032 |
| SGDS | 3.90 ± 0.44 | 3.91 ± 0.45 | 0.008 | 4.00 ± 0.43 | 4.32 ± 0.45 | 0.318 | 0.568 |
| GDS | 3.42 ± 0.07 | 3.48 ± 0.07 | 0.059 | 3.49 ± 0.07 | 3.60 ± 0.07 | 0.111 | 0.482 |
| MoCA-K | 17.07 ± 0.58 | 16.36 ± 0.59 | -0.711 | 15.72 ± 0.57 | 15.29 ± 0.58 | -0.433 | 0.594 |
| QoL-AD | 32.08 ± 0.71 | 31.89 ± 0.73 | -0.195 | 31.95 ± 0.70 | 32.25 ± 0.73 | 0.303 | 0.595 |
| NPI Total | 5.10 ± 1.45 | 5.60 ± 1.49 | 0.495 | 7.74 ± 1.43 | 11.06 ± 1.49 | 3.319 | 0.125 |
| FQoL-D | 92.15 ± 1.30 | 89.73 ± 1.35 | -2.418 | 94.20 ± 1.28 | 96.52 ± 1.34 | 2.319 | 0.015 |

Values are mean ± SE. Δ is computed as group mean at 26 weeks minus group mean at baseline. P-values for secondary/exploratory outcomes are nominal and are not adjusted for multiplicity; corresponding 95% confidence intervals and standardized effect sizes are provided to support estimation-focused interpretation.

K-MMSE, Korean Mini-Mental State Examination; CDR, Clinical Dementia Rating; K-IADL, Korean Instrumental Activities of Daily Living; SVLT-E, Seoul Verbal Learning Test–Elderly; RCFT, Rey Complex Figure Test; K-CWST, Korean Color-Word Stroop Test; DST, Digit Span Test; COWAT, Controlled Oral Word Association Test; K-BNT, Korean Boston Naming Test; SGDS, Short Geriatric Depression Scale; GDS, Global Deterioration Scale; MoCA-K, Montreal Cognitive Assessment–Korean; QoL-AD, Quality of Life in Alzheimer’s Disease; K-NPI, Korean Neuropsychiatric Inventory; FQoL-D, Family Quality of Life–Dementia; tDCS, transcranial direct current stimulation.
