# Supplementary material for: Six-month randomized, double-blind trial of transcranial direct current stimulation in mild Alzheimer's dementia: domain-specific cognitive and neuropsychiatric signals
Source: Front Neurol. 2026 Feb 23;17:1749559. doi: 10.3389/fneur.2026.1749559 (PMC12967943; doi:10.3389/fneur.2026.1749559)
Supplement: Supplementary file 2 [file Table_2.docx]

# **Supplementary Table 2. Outcomes at Baseline (0 week) and 26 Weeks — Per-Protocol (PP) Set**



| Endpoint | Active tDCS  0 week | Active tDCS  26 week | tDCS Δ | Sham tDCS 0 week | Sham tDCS 26 week | Sham Δ | p (Δ) |
| --- | --- | --- | --- | --- | --- | --- | --- |
| K-MMSE | 22.31±2.40 | 21.47±3.88 | -0.84 | 22.00±2.55 | 21.68±4.02 | -0.32 | 0.378 |
| CDR | 0.53 ± 0.12 | 0.64 ± 0.23 | +0.11 | 0.56 ± 0.16 | 0.68 ± 0.32 | +0.12 | 0.903 |
| K-IADL | 5.31 ± 3.31 | 6.31 ± 4.84 | +1.00 | 5.53 ± 3.96 | 6.91 ± 4.65 | +1.38 | 0.564 |
| SVLT-E | 3.50 ± 1.52 | 3.00 ± 1.61 | -0.50 | 3.53 ± 1.56 | 3.18 ± 1.55 | -0.35 | 0.672 |
| RCFT | 3.19 ± 4.83 | 4.95 ± 7.27 | +1.77 | 3.09 ± 3.94 | 2.85 ± 4.16 | -0.24 | 0.132 |
| K-CWST | 52.12±26.94 | 53.09±32.97 | +0.97 | 42.71±25.68 | 38.91±29.99 | -3.79 | 0.314 |
| DST | 5.12 ± 1.21 | 5.34 ± 1.15 | +0.22 | 5.03 ± 1.03 | 4.97 ± 1.00 | -0.06 | 0.174 |
| COWAT | 10.12 ± 4.01 | 9.09 ± 3.40 | -1.03 | 8.91 ± 4.60 | 8.03 ± 4.04 | -0.88 | 0.843 |
| K-BNT | 38.16±11.40 | 38.81±11.01 | +0.65 | 33.32±13.74 | 31.29±14.51 | -2.03 | 0.043 |
| SGDS | 4.06 ± 3.53 | 4.12 ± 3.83 | +0.06 | 3.15 ± 2.80 | 3.50 ± 3.51 | +0.35 | 0.617 |
| GDS | 3.41 ± 0.50 | 3.44 ± 0.50 | +0.03 | 3.53 ± 0.61 | 3.68 ± 0.68 | +0.15 | 0.212 |
| MoCA-K | 17.22 ± 3.93 | 16.28 ± 5.23 | -0.94 | 15.50 ± 4.67 | 14.74 ± 5.12 | -0.76 | 0.800 |
| QoL-AD (SUM1) | 31.81 ± 6.48 | 32.00 ± 6.38 | +0.19 | 33.50 ± 6.22 | 33.29 ± 6.09 | -0.21 | 0.746 |
| K-NPI Total (FRQ×GR1) | 6.38 ± 10.08 | 11.41±17.06 | +5.03 | 5.53± 8.75 | 5.06 ± 6.30 | -0.47 | 0.028 |
| FQoL-D | 91.16 ± 8.95 | 88.19±16.13 | -2.97 | 94.94±10.64 | 98.12±11.13 | +3.18 | 0.042 |

Values are mean ± SD. Δ is computed as group mean at 26 weeks minus group mean at baseline.

p is Welch t-test for Δ difference between groups. Group labels: Active tDCS vs Sham tDCS.

Per-endpoint PP sample sizes (n) by group:

- K-MMSE: Active n=34, Sham n=32

- CDR: Active n=34, Sham n=32

- K-IADL: Active n=34, Sham n=32

- SVLT-E: Active n=34, Sham n=32

- RCFT: Active n=34, Sham n=32

- K-CWST: Active n=34, Sham n=32

- DST: Active n=34, Sham n=32

- COWAT: Active n=34, Sham n=32

- K-BNT: Active n=34, Sham n=32

- SGDS: Active n=34, Sham n=32

- GDS: Active n=34, Sham n=32

- MoCA-K: Active n=34, Sham n=32

- QoL-AD (SUM1): Active n=34, Sham n=32

- K-NPI Total (FRQ×GR1): Active n=34, Sham n=32

- FQoL-D: Active n=34, Sham n=32- K-NPI Total (FRQ×GR1): Sham n=34, Active n=32

- FQoL-D: Sham n=34, Active n=32
