# Supplementary figures and images for: Six-month randomized, double-blind trial of transcranial direct current stimulation in mild Alzheimer's dementia: domain-specific cognitive and neuropsychiatric signals
Source: Front Neurol. 2026 Feb 23;17:1749559. doi: 10.3389/fneur.2026.1749559 (PMC12967943; doi:10.3389/fneur.2026.1749559)

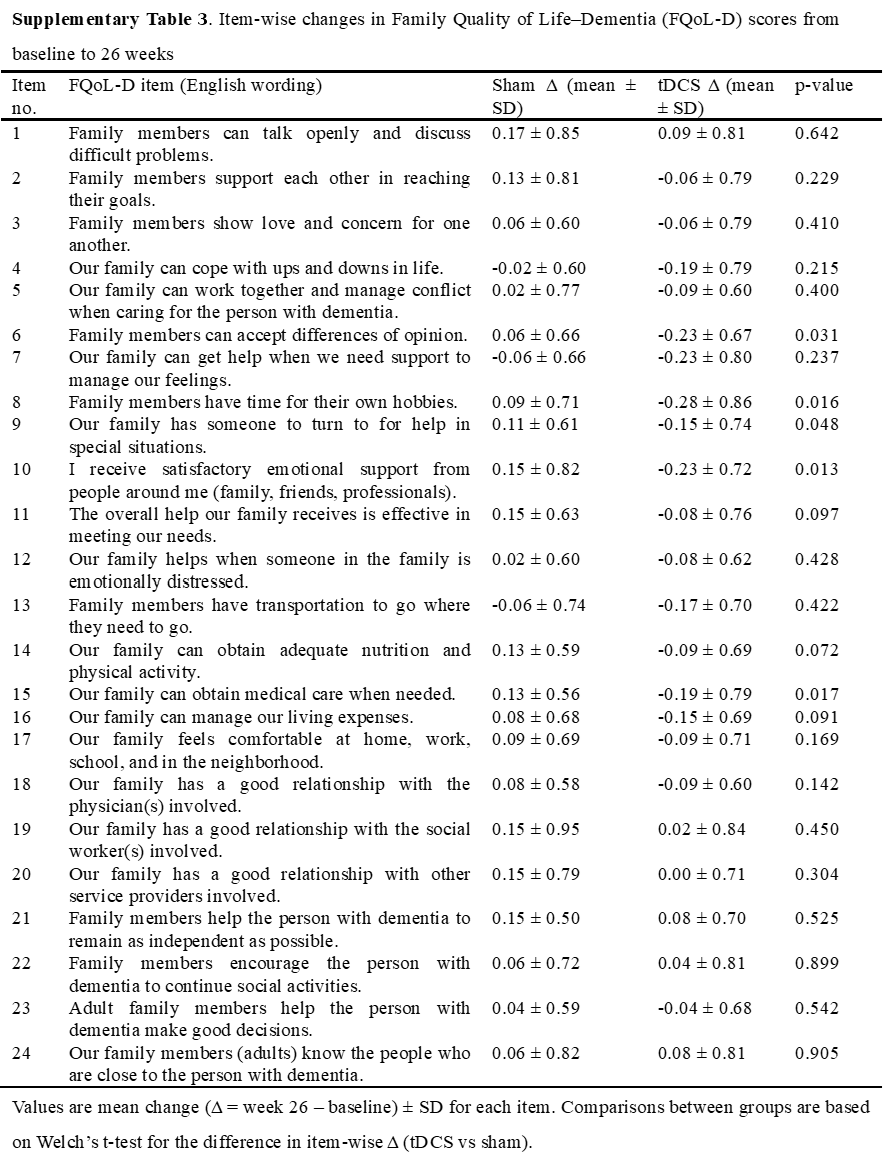


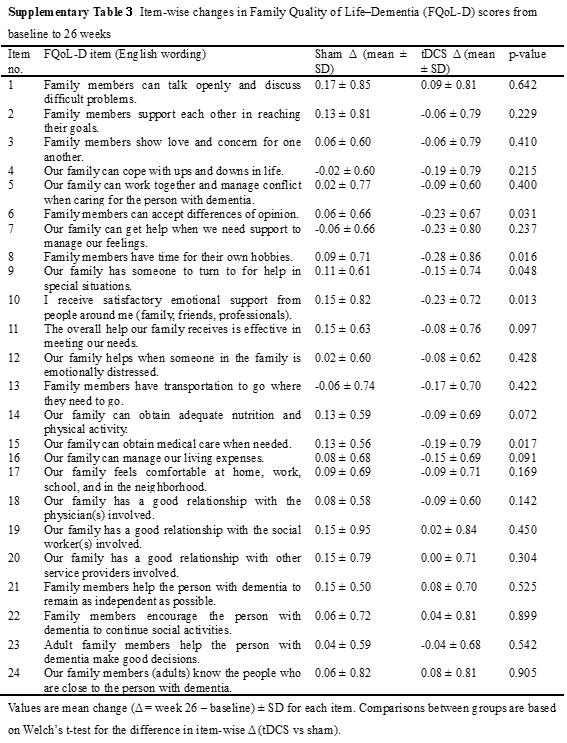

Supplement: Supplementary file 3 [file Table_3.docx]
