# Supplementary material for: Six-month randomized, double-blind trial of transcranial direct current stimulation in mild Alzheimer's dementia: domain-specific cognitive and neuropsychiatric signals
Source: Front Neurol. 2026 Feb 23;17:1749559. doi: 10.3389/fneur.2026.1749559 (PMC12967943; doi:10.3389/fneur.2026.1749559)
Supplement: Supplementary file 4 [file Table_4.docx]

**Supplementary Table 4. Adjusted between-group differences and standardized effects for key endpoints (ANCOVA)**

| **Endpoint** | **Adjusted diff (Active–Sham)** | **95% CI (diff)** | **p** | **Hedges’ g** | **95% CI (g)** |
| --- | --- | --- | --- | --- | --- |
| K-MMSE | -0.357 | -1.220 to 0.505 | 0.413 | -0.160 | -0.546 to 0.226 |
| K-BNT | 2.940 | 0.496 to 5.385 | 0.019 | 0.475 | 0.080 to 0.870 |
| FQoL-D | -5.419 | -9.397 to -1.442 | 0.008 | -0.534 | -0.925 to -0.142 |

Adjusted effects were estimated using ANCOVA at week 26 with baseline score and site as covariates (site fixed effects). Standardized effects are Hedges’ g based on the model residual SD. Analyses were restricted to the manuscript complete-case set defined by observed baseline and week-26 K-MMSE (n = 106; Active 53; Sham 53).

**Notes:** This table is provided to support estimation-focused interpretation for key endpoints (primary K-MMSE and nominal signals K-BNT and FQoL-D). P-values for secondary/exploratory outcomes are nominal and not adjusted for multiplicity. Supplementary Table 2 reports covariate-adjusted estimates from ANCOVA (baseline score and site as fixed effects); thus, estimates and p-values may differ slightly from unadjusted change-score comparisons in Table 2
